# Dr. Leonidas Franklin Wilbur

**Published:** 1911-10

**Authors:** 


Dr. Leonidas Franklin Wilbur, of Honeoye, died September 2, 1911, aged 79 years. He graduated at Harvard in 1854. For over 50 years, he had been a member of the Medical Society of the County of Ontario.

